# Hydroxypropyl-β-Cyclodextrin-Based *Helichrysum italicum* Extracts: Antioxidant and Cosmeceutical Activity and Biocompatibility

**DOI:** 10.3390/antiox12040855

**Published:** 2023-04-01

**Authors:** Lejsa Jakupović, Ivana Bačić, Jasna Jablan, Eva Marguí, Marijan Marijan, Suzana Inić, Laura Nižić Nodilo, Anita Hafner, Marijana Zovko Končić

**Affiliations:** 1 Department of Pharmacognosy, University of Zagreb Faculty of Pharmacy and Biochemistry, A. Kovačića 1, 10000 Zagreb, Croatia; 2 Forensic Science Centre “Ivan Vučetić”, Ilica 335, 10000 Zagreb, Croatia; 3 Department of Analytical Chemistry, University of Zagreb Faculty of Pharmacy and Biochemistry, A. Kovačića 1, 10000 Zagreb, Croatia; 4 Department of Chemistry, Faculty of Sciences, University of Girona, C/M. Aurèlia Capmany 69, 17003 Girona, Spain; 5 Department of Pharmaceutical Technology, University of Zagreb Faculty of Pharmacy and Biochemistry, A. Kovačića 1, 10000 Zagreb, Croatia

**Keywords:** anti-inflammatory, antioxidant, cosmeceutical, HaCaT cells, *Helichrysum italicum*, hyaluronidase

## Abstract

Two *Helichrysum italicum* extracts, OPT-1 (rich in phenolic acids) and OPT-2 (rich in total phenols and flavonoids), were prepared using hydroxypropyl-β-cyclodextrin (HP-β-CD)-assisted extraction. The prepared extracts were rich in phenolic compounds, including flavonoids and phenolic acids. GC-MS analysis of the extracts identified neryl acetate, neo-intermedeol, β-selinene, γ-curcumene, italidione I, and nerol as the main volatile components of the extracts, as well as plant sterols, γ-sitosterol, campesterol, and stigmasterol. The antioxidant (DPPH radical scavenging, reducing power, and a carotene linoleic acid assay) and cosmeceutical (anti-hyaluronidase, anti-tyrosinase, anti-lipoxygenase, ovalbumin anti-coagulation, and a UV-absorption assay) activity of the extracts in most of the assays was better than the activity of the applied positive controls. Especially low were the IC_50_ values of the extracts in the anti-hyaluronidase (14.31 ± 0.29 μL extract/mL and 19.82 ± 1.53 μL extract/mL for OPT-1 and OPT-2, respectively) and the anti-lipoxygenase (0.96 ± 0.11 μL extract/mL and 1.07 ± 0.01 μL extract/mL for OPT-1 and OPT-2, respectively) assays. The extracts were non-toxic to HaCaT cells in concentrations of up to 62.5 µL extract/mL assuring their status as excellent candidates for cosmeceutical product development appropriate for direct use in cosmetic products without solvent evaporation.

## 1. Introduction

As the importance of both health and physical appearance is gaining more significance, the cosmetic industry is exploring new strategies for development to fulfill consumers’ needs and expectations. For achieving this goal, the term “cosmeceuticals” has been coined to describe the products that are presumed to have the combined characteristics of cosmetics and medication. Such products may be considered functionalized cosmetics that exhibit therapeutic effects along with intended cosmetic effects. Even though the word “cosmeceutical” is not a legal term, it is often used by consumers and professionals alike because it connects a cosmetic product that beautifies and pharmaceutical products that cure certain diseases [1]. Among cosmeceuticals, the ingredients obtained from plants are in special demand, due to the consumers inclination for natural skin-care and their beneficial effects on skin [2]. Indeed, numerous studies have shown that ingredients of natural origin may impede processes that adversely influence skin health and appearance, for example, by acting as antioxidants or by defending the skin’s macromolecules against enzymatic degradation caused by aging or exposure to environmental factors [3].

*Helichrysum italicum* (Roth) G. Don, Asteraceae (immortelle), is a plant widely spread in the Mediterranean region that is traditionally used for digestive and respiratory ailments. The plant is also used for the topical treatment of skin inflammation and infections [4]. *H. italicum* is highly valued for its essential oil and is particularly rich in neryl acetate and *α*-pinene. The oil also contains α- and *γ*-curcumene, *α*-and *β*-selinene, italidione I and II, nerol, limonene, and linalool [5]. The most important among the non-volatile secondary metabolites are the hydroxycinnamic acid derivatives (rosmarinic, neochlorogenic, isochlorogenic B, cichoric [6], 3,4-dicaffeoylquinic, chlorogenic, and 3,5-dicaffeoylquinic acid [7]), hydroxybenzoic acids (e.g., 3,4-dihydroxybenzoic, 2,4-dihydroxybenzoic, and vanillic acid) [8], and flavonoids (rutin [6], tiliroside [8], and kaempferol 3-*O*-glucopyranoside [7]). In addition, *H. italicum* contains pyrone derivatives, with arzanol being the most important one [8] due to the many biological activities that it displays. 

The use of *H. italicum* in cosmetics is growing rapidly as it is widely presumed that it can delay or even prevent skin aging [6]. Scientific studies have confirmed that *H. italicum* extracts and its essential oil, as well as the phenolic and other substances that the plant contains, display antioxidant and antibacterial activity [6], the ability to decrease skin irritation [9], inhibit the enzymes that adversely affect skin appearance, such as elastase and collagenase [5,10], and even accelerate wound healing in vivo [11]. Arzanol, for example, has been identified as the major anti-inflammatory and anti-viral constituent from *H. italicum*. It inhibits releases of interleukins IL-1 β, IL-6, and IL-8 and tumor necrosis factor (TNFα) [12]. Arzanol inhibits 5-lipoxygenase (LOX) activity, as well as the activity of cyclooxygenase (COX)-1 and the biosynthesis of COX-2-derived prostaglandin E2 (PGE₂) [13]. Antioxidant activity of arzanol has been demonstrated in numerous in vitro models [14]. Other phenolic plant constituents, e.g., flavonoids, such as luteolin and apigenin derivatives, display anti-erythematous and photoprotective activities [4]. In addition, it has been repeatedly shown that *H. italicum* essential oil [6,15,16] and hydrosol [16] display antimicrobial and antioxidant properties.

Before being incorporated into cosmetic formulations, secondary plant metabolites must first be extracted from plant material. Ideally, this is achieved using solvents that, besides having high dissolving properties, should be safe, both to human organisms and the environment [17,18]. Among such solvents, aqueous solutions of cyclodextrins (CDs), cyclic oligosaccharides made up of six (α-CDs), seven (β-CDs), or eight (γ-CDs) d-glucopyranoside units linked by α-1,4-glycosidic bonds [19], have a significant place. CD molecules have a cyclic structure with a hydrophobic interior and a hydrophilic exterior that enables them to form complexes with small molecules (e.g., flavonoids and other plant phenolics). This enhances their water solubility and stability, the features that are important for the formulation of different cosmetic and pharmaceutical products [19]. One of the additional advantages of CDs use in cosmetics is that they may enhance the bioavailability [20] and transport of active molecules through the epidermis [21]. The addition of polar side chains, such as additional hydroxypropyl group, may enhance the solubility of CDs in water such as, for example, in (2-hydroxypropyl)-β-cyclodextrin (HP-β-CD). Such structural features can additionally increase the stability of CD–flavonoid complexes as exemplified in HP-β-CD encapsulation complexes with rutin [20], or kaempferol derivatives [22,23]. In addition, the use of HP-β-CD was proven to be an effective strategy for the encapsulation of volatile organic molecules such as carvacrol, cinnamaldehyde, eugenol, limonene, linalool, thymol [24], and α-pinene [25]. 

In our previous study, exhaustive extraction optimization of the HP-β-CD-assisted extraction of *H. italicum*, as well as its comparison with extraction using conventional solvents, such as water and ethanol, was presented. It was found that the presence of HP-β-CD greatly increases the efficiency of extraction. In the previous study, two extracts were prepared using the optimized procedure: OPT-1, having high total phenolic acid content (TPA), and OPT-2, high in total phenol (TP) and total flavonoid (TF) content. The extracts were rich in bioactive phenolic compounds, especially arzanol and 3,5-dicaffeoylquinic acid. Furthermore, they presented potent anti-elastase and anti-collagenase properties [10]. The summary of the performed research is presented in Table S1. In continuation of the study of HP-β-CD-based extracts, further analysis of the chemical composition of the two extracts, as well as a thorough investigation of their cosmeceutical activity and biocompatibility was performed with the aim of obtaining highly active compounds and ingredients suitable for direct use in cosmetic products.

## 2. Materials and Methods

### 2.1. Chemicals

Butylated hydroxyanisole (BHA ≥ 98.5%), hyaluronidase from bovine testes, diclofenac (≥98%), nordihydroguaiaretic acid (NDGA ≥ 98.5%) kojic acid (≥98.5%), tannic acid, 3-(4,5-dimethylthiazol-2-yl)-2,5-diphenyltetrazolium bromide, and mushroom tyrosinase were purchased from Sigma-Aldrich (St. Louis, MO, USA). Soybean LOX was a product from TCI chemicals (Tokyo, Japan). The spontaneously immortalized human keratinocyte line (HaCaT) was purchased from (CLS Cell Line Services, Heidelberg, Germany). The buffers and chemicals used for cell cultivation were as follows: Hank’s balanced salt solution (HBSS; pH 6.0) (Capricorn Scientific, Ebsdorfergrund, Germany), Dulbecco’s modified Eagle medium (DMEM) (Sigma-Aldrich, St. Louis, MO, USA), FBS (Biosera, Boussens, France), penicillin, streptomycin, and amphotericin B (5%, Lonza, Basel, Switzerland). The other reagents and chemicals were of analytical grade.

### 2.2. Apparatus

For ultrasound-assisted extraction, a SONOREX^®^ Digital 10 P DK 156 BP ultrasonic bath (Bandelin, Berlin, Germany) was used. Spectrophotometric determinations were performed using a 1420 Multilabelcounter VICTOR3 microplate reader (PerkinElmer, Waltham, MA, USA) (cell viability assay) and a FLUOstar Omega (BMG Labtech, Ortenberg, Germany) microplate reader (other assays). For phase-contrast microscopy, a Primovert microscope (Carl Zeiss AG, Oberkochen, Germany) was used. The microwave oven used for digestion was a Speedwave XPERT (Berghof products & Instruments GmbH, Eningen unter Achalm, Baden-Württemberg Germany). An Agilent 7500c model ICP-MS system (Agilent Technologies, Santa Clara, CA, USA) equipped with an octapole collision cell was used for simultaneous multielement detection in the plant material. Gas chromatography–mass spectrometry analysis was carried out on a GC-MS-QP2010 Ultra system (Shimadzu, Kyoto, Japan).

### 2.3. Plant Material

*H. italicum* was collected in Zadar County, Croatia (44°00′28″ N; 15°32′23″ E), in June 2020. The species was collected and its identity wasestablished by Suzana Inić. A voucher specimen (HI-2020-6-1) has been deposited in the plant collection of the Department of Pharmacognosy, Faculty of Pharmacy and Biochemistry, University of Zagreb (Croatia). Prior to use, fresh flowering aerial parts of *H. italicum* were milled and sieved through an 850 μm mesh size sieve.

### 2.4. Sample Preparation for the Determination of Metal Content

To prepare the plant samples, a microwave acid digestion United States Environmental Protection Agency (EPA) method 3052 [26] was used. The sample (250 mg) was mixed with nitric acid (9 mL) and H_2_O_2_ (1 mL) in a PTFE vessel that was consequently sealed and heated first for 5 min until reaching 180 °C and then for 10 min at 180 °C. Upon cooling, the remaining mixtures were transferred to a 25-mL flask and made up with ultrapure water.

### 2.5. Inductively Coupled Plasma Mass Spectrometry (ICP-MS)

For simultaneous detection of Se, As, Cr, Ni, Fe, Cu, Zn, Sr, and Pb in the plant material, inductively coupled plasma mass spectrometry (ICP-MS) was used. The instrumental parameters were as follows: the RF power was 1500 W, the plasma gas flow rate was 15 L min^−1^, and the nebulizer gas flow rate was 1.2 L min^−1^. The sampling cone was made of Ni with a 1 mm aperture diameter, while the skimmer cone was made of Ni with a 0.4 mm aperture diameter. The integration time for each isotope was 0.1 s. Three readings per replicate were performed. For a particular isotope, different cell conditions (in brackets) were used: ^208^Pb and ^103^Rh (without gas), ^75^As, ^78^Se, and ^103^Rh (H_2_ 3 mL/min and He 0.5 mL/min), and ^53^Cr, ^56^Fe, ^60^Ni, ^63^Cu, ^66^Zn, ^88^Sr, and ^103^Rh (He 2 mL/min). To obtain quantitative results, ^103^Rh was used as an internal standard. Matrix-matched calibration standards were used for quantification.

### 2.6. Preparation of the Extract

Two *H. italicum* extracts were prepared, OPT-1 and OPT-2 (formerly OPT-1 and OPT-2), according to a previously published protocol [10]. In short, fresh powdered plant material (0.89 g) and HP-β-CD (0.6 mmol) were quickly dispersed in 10 g of 1.95%, *w*/*w* lactic acid (OPT-1) or water (OPT-2) and quickly stirred. Following that, the flask was placed in an ultrasonication bath at 144 W ultrasonication power at 80 °C for 30 min. The extracts were filtered and kept at −20 °C before use.

### 2.7. Spectrophotometric Determination of Total Phenolic Content

For TP content determination [27], 80 μL of each extract, Folin–Ciocalteu reagent, and 10% *w*/*w* sodium carbonate solution were mixed. After 1 h, the absorbance at 700 nm was recorded. The TP concentration was determined from the calibration curve of gallic acid and expressed as mg of gallic acid per mL of extract.

### 2.8. Spectrophotometric Determination of Total Phenolic Acid Content

For TPA determination [28], 0.5 M HCl (50 μL), nitrite molybdate reagent (prepared from 10 g of NaNO_2_ and 10 g of Na₂MoO₄ made up to 100 mL with distilled water), 8.5% NaOH (50 μL), and the extract (100 μL) were added. The TPA content was calculated from the calibration curve of caffeic acid and expressed as mg of caffeic acid per mL of extract.

### 2.9. Spectrophotometric Determination of Total Flavonoid Content

The TF content was determined by mixing the extract (120 μL) and 0.2% *w*/*w* AlCl_3_ methanolic solution (120 μL) [29] and measuring the absorbance (420 nm) after 1 h. The TF content was calculated from the calibration curve of quercetin and expressed as mg of quercetin per mL of extract.

### 2.10. GC-MS Analysis

To prepare the sample for GC-MS analysis, 3 mL of the extracts was extracted with 3 mL cyclohexane. The upper layer was dried over anhydrous Na_2_SO_4_. Upon filtration, the cyclohexane solution was stored at −20 °C in the dark until use. The samples (1 μL) were automatically injected with a split ratio 20:1 and separated on an HP-1 MS capillary column (30 m × 0.25 mm × 0.25 µm) (Agilent, Santa Clara, CA, USA). The flow rate of He as a carrier gas was 1 mL/min, while the injector temperature was set at 270 °C. The oven settings were: the initial temperature was held at 45 °C for 3.7 min, then increased to 295 °C at 10 °C/min, and held at this temperature for 10 min. The mass spectrometer (electron impact at 70 eV) scanned the mass range 45–550 *m*/*z*. The interface temperature was 295 °C, and the ion source temperature was 230 °C. The solvent delay time was 3.7 min while the total run time for the sample analysis was 38.7 min. Tentative compound identification was achieved by comparison of their mass spectra with the mass spectra stored in NIST17 and Wiley 275 libraries. Deconvolution of partially resolved peaks and compound relative percentage calculation in total ion chromatograms (TIC) was performed by LabSolutions GCMS software version 4.45 SP1.

### 2.11. Radical Scavenging Activity

To estimate radical scavenging activity (RSA), a 2,2-diphenyl-1-picrylhydrazyl (DPPH)-free radical was used according to a previously published method [30]. To a solution of 130 μL of the extract in methanol, DPPH solution (70 μL, 0.21 mg/mL) was added. After 30 min, the absorbance was recorded at 545 nm. RSA was calculated according to Equation (1):

(1) $$\mathrm{RSA}\;\left(\%\right)=\frac{A_{0}-A_{s}}{A_{0}}\times 100$$

where A_0_ is the absorbance of the negative control which used methanol instead of the extract and A_s_ is the absorbance of the respective extract. The concentration of the extract which scavenges 50% of free radicals present in the solution (RSA IC_50_) was calculated. BHA was used as the positive control.

### 2.12. Antioxidant Activity in the β-Carotene-Linoleic Acid Assay

The antioxidant activity in the β-carotene-linoleic acid assay was evaluated in accordance with a modified literature procedure [31]. An emulsion containing β-carotene (0.75 mg/mL), linoleic acid (1.1 mg/mL), and Tween 40 (8.5 mg/mL) was prepared. An aliquot of 200 μL of the emulsion was pipetted to a microtiter well containing the extract solution in methanol (50 μL). The reaction mixture was incubated at 50 °C. The activity in the β-carotene linoleic acid assay (ACL) was calculated based on the absorbances recorded at the beginning of the reaction and after 60 min using Equation (2):

(2) $$\mathrm{ACL}\;\left(\%\right)=\frac{A_{\mathrm{sample}\;\left(t=60\right)}\;}{A_{\mathrm{control}\;\left(t=0\right)}\;}\times 100$$

where A_control_ and A_sample_ are the absorbances of the methanol control and the extract, respectively. The concentration of the extract that protects 50% β-carotene present in the solution (ACL IC_50_) was calculated. The BHA was used as the positive control.

### 2.13. Reducing Power

The reducing power of the extracts was determined according to a previously used method [32] modified for the microtiter plate. Briefly, the extract solution (40 µL) was mixed with water (40 µL), phosphate buffer (0.2 M, pH 6.6, 100 µL), and potassium ferricyanide (1%. *w*/*w*, 100 µL). After 20 min of incubation at 50 °C, trichloroacetic acid (10% *w*/*w*, 100 µL) was added to the mixture. Upon 10 min of centrifugation (3000 rpm), an aliquot of 125 µL of the supernatant was transferred to a new plate. The mixture was two-fold serially diluted. Following that, 25 µL of a 0.1% (*w*/*v*) ferric chloride solution was added and the absorbance read spectrophotometrically at 700 nm. The concentration of the extract that achieved the absorbance of 0.5 at 700 nm (RP EC_0.5_) was calculated. Ascorbic acid was used as the positive control.

### 2.14. Hyaluronidase Inhibitory Activity

For anti-hyaluronidase activity determination [33], the extract solution (25 µL) and hyaluronidase solution (20 µL, 4 mg/mL) were mixed and incubated at 37 °C. After 20 min, CaCl_2_ solution (40 µL, 12.5 mM) was added and the resulting mixture was further incubated at the same temperature. After an additional 20 min, 50 µL of sodium hyaluronate solution (3.5 mg/mL) was added and the reaction mixture was incubated for 40 min at 37 °C with constant shaking. Following that, the reaction was stopped by the addition of NaOH (20 µL, 0.9 M) and sodium tetraborate (40 µL, 0.2 M). The mixture was then quickly heated at 100 °C for 3 min. Following that, 160 µL of *p*-dimethylaminobenzaldehide reagent (DMABA) (0.25 g DMABA dissolved in 4.4 mL of acetic acid and 0.6 mL of 10 M HCl) was added. The mixture was incubated again at 37 °C. After 10 min, the absorbance at 585 nm was measured. Tannic acid was used as the positive control. The hyaluronidase inhibitory activity (HyalInh) was calculated as shown in Equation (3):

(3) $$\mathrm{HyalInh}\;\left(\%\right)=\frac{A_{0}-A_{s}}{A_{0}}\times 100$$

Where A_0_ is the absorbance of the negative control (the solution containing the buffer instead of the extract) and A_s_ is the absorbance of the corresponding extract. HyalInh IC_50_ was calculated as the concentration of the extract that inhibited 50% of hyaluronidase activity and was expressed as μL of extract/mL of solution.

### 2.15. Tyrosinase Inhibitory Activity

For tyrosinase inhibitory activity [34], the extract solution (80 μL) and 40 μL of tyrosinase solution in the phosphate buffer (16 mM pH 6.8) were mixed, and the reaction was left at room temperature in the dark. After 10 min, 80 μL of L-DOPA solution (0.19 mg/mL in phosphate buffer) was added. After 10 min, the resulting absorbance was measured at 492 nm. Tyrosinase inhibitory activity (TyInh) was calculated as in Equation (4):

(4) $$\mathrm{TyInh}\;\left(\%\right)=\frac{A_{0}-A_{s}}{A_{0}}\times 100$$

where A_0_ is the absorbance of the negative control (where the buffer was used instead of the extract) and A_s_ is the absorbance of the respective extract. The concentration of the extract which inhibits 50% of tyrosinase activity (TyInh IC_50_) was calculated. Kojic acid was used as the positive control.

### 2.16. Measurement of the UVA and UVB Absorbing Capabilities of H. italicum Extracts

The spectra of the extracts and *p*-aminobenzoic acid (PABA) solution (1 mg/mL), diluted in the water in a 1:64 ratio were recorded [35]. Absorbance was measured at wavelengths from 290 to 400 nm. Areas under curves (AUCs) were measured in two wavelength ranges: 290–320 nm (UVB) and 320–400 nm (UVA).

### 2.17. Lipoxygenase Inhibitory Activity

For LOX inhibitory activity [36], 25 μL of the LOX solution (0.0032 mg/mL), 100 μL of the extract solution, and 50 μL of the phosphate buffer (pH 8, 100 mM) were mixed. After 5 min, 50 μL of linoleic acid in the phosphate buffer (pH 8, 100 μM) was mixed and incubated at 25 °C. After 45 min, the absorbance was measured at 234 nm. The LOX inhibitory activity (LOXInh) was calculated as in Equation (5):

(5) $$\mathrm{LOXInh}\;\left(\%\right)=\frac{A_{0}-A_{s}}{A_{0}}\times 100$$

where A_c_ is the absorbance of the negative control (the reaction mixture containing the buffer solution instead of the extract) and A_s_ is the absorbance of the corresponding extract. LOXInh IC_50_ was calculated as the concentration of the extract that inhibited 50% of LOX activity and was expressed as μL of extract/mL of solution. NDGA was used as the positive control.

### 2.18. Inhibition of Heat-Induced Ovalbumin Coagulation

Inhibition of heat-induced ovalbumin coagulation [37] was evaluated as follows. The extract solution (80 μL) in phosphate-buffered saline (pH 7.4) and ovalbumin solution in the same buffer (90 μL) were quickly mixed and then incubated for 15 min at 37 °C. Following this, the solution was heated at 70 °C for 5 min. After cooling of the reaction mixture, the intensity of the haze was estimated by recording the absorbance at 660 nm. The inhibition of ovalbumin denaturation (OvInh) was calculated by using Equation (6):

(6) $$\mathrm{OvInh}\;\left(\%\right)=\frac{A_{0}-A_{s}}{A_{0}}\times 100$$

where A_0_ is the absorbance of the negative control (water) and A_s_ is the absorbance of the respective extract. The concentration of the extract which inhibits 50% of ovalbumin coagulation (OvInh IC_50_) was calculated. Diclofenac sodium was used as the positive control.

### 2.19. Cell Culture Conditions

The HaCaT cell line for the experiments was cultivated using DMEM supplemented with FBS (10%, *w*/*w*), penicillin, streptomycin, and amphotericin B (5%, *w*/*w*). The cells were passaged at 80–90% confluence. The medium was changed approximately every 48 h. The cultures were maintained at 95% humidity and 37 °C in an atmosphere of 5% CO_2_.

### 2.20. Cell Viability Study

For determination of cell viability, the 3-(4,5-dimethylthiazol-2-yl)-2,5-diphenyltetrazolium bromide test (MTT test) was used [38]; the HaCaT cells were seeded at a density of 2 × 10^4^ cells/well and incubated for 24 h to reach confluence. Following this, the cell culture medium was withdrawn, and the cells were washed with HBSS. The extracts were diluted with HBSS (pH 6.0) in concentrations of 12.5–125 μL/mL and the extract solutions were used to treat the cells. After 2 h, the cells were washed twice with HBSS and incubated with fresh medium (500 µL/well). After 24 h, 50 µL of the MTT solution (5 mg/mL) was added to each well and the plates were incubated for 1 h at 37 °C. Following that, the medium was removed, and the cells were lysed. The formazan formed in the reaction was dissolved with acidic isopropanol and the absorbance at 570 nm was measured. Metabolic activity was expressed as relative to the negative control (untreated cells incubated in HBSS).

### 2.21. Statistical Analysis

For evaluation of antioxidant and enzyme-inhibiting activity, the results were presented as the mean ± standard deviation of three measurements. IC_50_ values were calculated using regression analysis. Statistical comparisons were made using the Student’s *t*-test (GraphPad Prism) for comparison between the extracts, and the Dunnett’s post hoc test for was used for comparison with the control. *p*-values < 0.05 were considered statistically significant.

## 3. Results and Discussion

### 3.1. Determination of Metal Content in H. italicum Aerial Parts

Before the extraction of the *H. italicum* aerial parts, its suitability for preparation of topical products was assessed by determining not only the content of heavy metals, whose presence represents a potential safety hazard for consumers, but also the presence of metals which may exert a beneficial effect on the skin. Cosmetic preparations are repeatedly applied directly to human skin and mucous membranes, as well as skin appendices, hair, and nails. Therefore, not only their efficacy, but also their safety, is among users’ primary concerns. The toxic effects of cosmetics and other products applied on skin are sometimes related to the presence of chemical substances, including toxic metals [39]. Due to ever-increasing air and soil pollution, plant material is especially prone to the accumulation of toxic heavy metals from the environment. To identify the plant material best suited for further analysis, both the stem and the flower of *H. italicum* were analyzed. The results are presented in Table 1. The content of toxic heavy metals, chromium, lead, nickel, and arsenic in both samples was either low or non-detectable and thus significantly below the legal limits for cosmetic products [40]. Thus, even though there were quantitative differences in the mineral composition, regarding the heavy metal content, both the stem and the flower of *H. italicum,* were found to be appropriate for cosmetic preparations. As a consequence, all of the aerial parts were used for further study.

In addition to the product’s safety, metal ions in plant materials used for the preparation of cosmetics may affect the stability and efficacy of the cosmetic product. Therefore, the contents of selected metals and one non-metal (Se) in the plant material were determined (Table 1) and it was found that *H. italicum* contained several minerals that may beneficially affect the skin.

Copper and iron may have a dual influence on the safety, stability, and efficacy of topical products. In low doses, they are necessary for the functioning of the whole organism, including the skin, but harmful when they occur in excessive amounts [39]. Both metals were present in substantial amounts, especially in the flowers. Iron was the most represented metal in the investigated material. Iron in cosmetic products may play a dual role. It may support the beneficial skin-related effects of the product because it is essential for many processes such as wound healing, inflammatory response, and the synthesis of skin collagen. On the other hand, high concentrations of this metal adversely affect the stability of the product and shortens its shelf-life [27,41]. Therefore, when making cosmetic formulations, precautions should be taken to ensure that the stability of the products is ensured, e.g., by adding appropriate iron chelators. The investigated material contained a significant amount of copper, an element necessary for melanogenesis because two Cu^2+^ ions are present at the active site of the enzyme tyrosinase [42]. Copper modulates several cytokines and growth factor mechanisms of action and is essentially involved in all stages of the wound healing process. Furthermore, copper is important for skin regeneration because it accelerates the healing process through the induction of angiogenesis [43]. European Union law allows the presence of copper pigments in cosmetics [39]. However, caution should be exercised in excessive copper use because topically applied CuO nanoparticles have been reported to enhance the secretion of inflammatory cytokines and cause necrosis in human skin organ cultures [39]. 

Some minerals mostly display beneficial skin-related properties. Since *H. italicum* is traditionally used for the alleviation of skin disorders and minor wounds [9], the content of zinc, the metal that may support the skin-related properties of plant extracts, was also determined. Zinc was among the most represented metals in the investigated material, second only to iron. Zinc insufficiency impedes wound healing [44] as keratinocyte proliferation and differentiation are dependent on the presence of this metal [41]. In addition, the collected material contains small amounts of selenium, an essential trace element in the human body. Selenium contributes to the alleviation of reduced reactive-oxygen-species-mediated inflammation, reduced DNA damage, and prolonged telomere length. Therefore, it plays roles in preventing aging and aging-related disorders, including skin aging [45]. Selenium is important for cellular anti-oxidant and anti-UV radiation defense [46], while selenium sulfide is used in the treatment of seborrheic dermatitis [47]. Finally, the plant material, and especially the stalks, contained substantial amounts of strontium (Table 1), a metal whose salts (e.g., strontium chloride hexahydrate) are often used in stomatology as they reduce the sensitivity of the gums in periodontal disease as they reduce inflammation and TNF-α levels [48].

### 3.2. Phenolic Content of the Extracts

Plant phenolics, including phenolic acids and flavonoids, are among the most investigated plant ingredients in cosmetic products due to their ability to display various biological activities that suppress aging such as antioxidant activity, the inhibition of dermal proteases, and photoprotective activity [49]. Flavonoids are a ubiquitous class of polyphenols, widely distributed in the aerial parts of terrestrial plants. Due to their chemical structure, they display distinctive antioxidant activity. They may protect both the skin and the cosmetic product from detrimental free radicals and UV radiation [50]. In addition, they act as depigmentation agents [51]. Similar to flavonoids, phenolic acids display strong antioxidant properties, as well as displaying photoprotective, anti-inflammatory, and depigmenting effects. In addition, they may reduce the activity of matrix-degrading enzymes such as collagenase and thus act as anti-aging agents [52]. 

**Figure 1:**
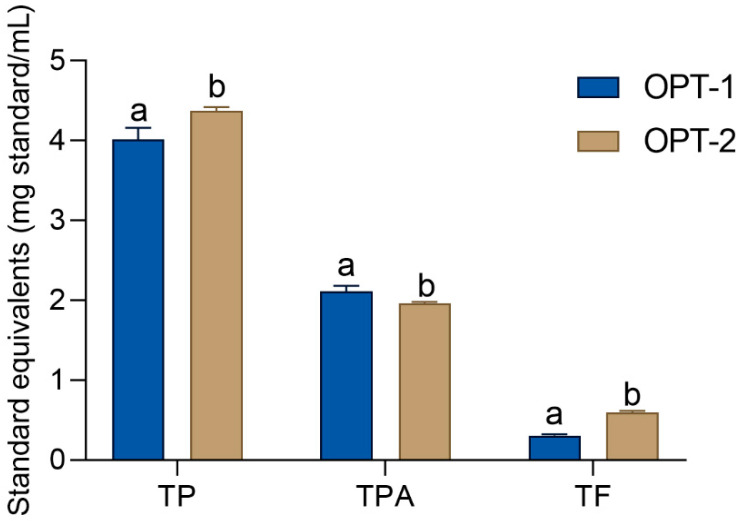
Total phenolic (TP), total flavonoid (TF), and total phenolic acid (TPA) content of the OPT-1 and OPT-2 extracts (prepared as described in Section 2.6). The standards equivalents for the determination of TP, TPA, and TF were gallic acid, caffeic acid, and quercetin, respectively. Different lowercase letters indicate statistically significant difference between the phenols of the same type (grouped together) (Student’s *t*-test, *p* < 0.05). The TPA in OPT 1 as well as the TP and TF in OPT 2 have been previously published in [10].

The TP, TPA, and TF content of the extracts are presented in Figure 1. While TPA in OPT 1, as well as TP and TF in OPT 2, have been previously published in [10] (Table S1), this research provides new information on TP and TF in OPT 1, as well as TPA in OPT 2. In our previous work, we optimized an HP-β-CD-assisted procedure for preparation of an extract containing a high amount of phenolic compounds. The exhaustive extraction optimization, reported in the previous study [10], showed that a small amount (1.95%, *w*/*w*) of lactic acid positively contributed to TPA extraction, while its presence exerted a negative influence on TP and TF extraction. As a result, two extracts were prepared using an optimized HP-β-CD-assisted procedure: OPT-1, with high TPA and prepared using lactic acid, while for the preparation of OPT-2, the extract high in TP and TF, lactic acid was not added. As can be observed in Figure 1, both extracts were rich in phenolic compounds. Expectedly, OPT-1 contained more TPA, and OPT-2 was richer in TP and TF. The previously performed and reported [10] LC-MS analysis of OPT-1 and OPT-2 showed that the main metabolites in both extracts were the derivatives of hydroxycinnamic acid, especially caffeoylquinic acid derivatives of which 3,5-*O*-dicaffeoylquinic acid was present in the highest amount. Another highly represented group were flavonols, such as the derivatives of quercetin, myricetin, isorhamnetin, and kaempferol. In addition, both extracts contained arzanol and 3-methylarzanol. Interestingly, the addition of lactic acid exerted the positive influence of arzanol extraction, so OPT-1 was richer in that phloroglucinol α-pyrone [10].

### 3.3. GC-MS Analysis

**Figure 2:**
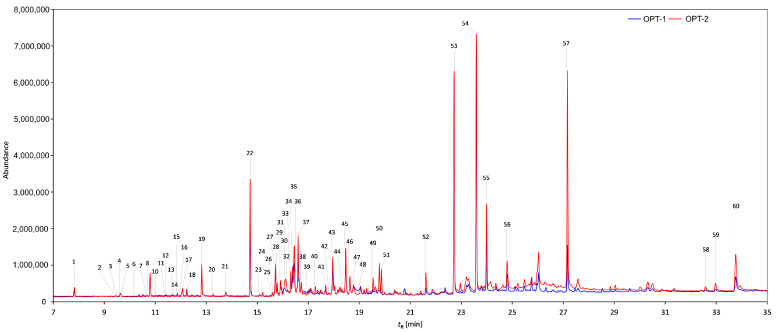
GC-MS chromatograms of the OPT-1 (blue line) and OPT-2 (red line) extracts (prepared as described in Section 2.6). The tentative identification is as follows: **1** = α-pinene, **2** = α-terpinene, **3** = p-cymene, **4** = eucalyptol, 5 = d-limonene, **6** = γ-terpinene, **7** = cyclooctanone, **8** = α-terpinolene, **9** = linalool, **10** = fenchol, **11** = camphor (2-bornanone), **12** = l-pinocarveol, **13** = 2-methylbutyl angelate, **14** = nerol oxide, **15** = endo-borneol (Camphol), **16** = 4,6-dimethyloctane-3,5-dione, **17** = 4-terpineol, **18** = α-terpineol, **19** = nerol, **20** = linalylacetate, **21** = 4-hydroxy-3-methylacetophenone, **22** = neryl acetate, **23** = α-muurolene, **24** = α-copaene, **25** = β-curcumene, **26** = trans-α-bergamotene, **27** = 4,6,9-trimethyldec-8-en-3,5-dione, **28** = β-caryophyllene, **29** = neryl propionate, **30** = cis-α-bergamotene, **31** = *n.i.*, **32** = humulene, **33** = 2,4,6,9-tetramethyldec-8-en-3,5-dione, **34** = α-curcumene, **35** = γ-curcumene, **36** = β-sesquisabinene, **37** = β-selinene, **38** = γ-selinene, **39** = d-cadinene, **40** = dodecanoic acid (lauric acid), **41** = caryophyllene oxide, **42** = guaiol, **43** = (1S,3αS,4S,5S,7αR,8R)-5-Isopropyl-1,7α-dimethyloctahydro-1H-1,4-methanoinden-8-ol, **44** = t-cadinol, **45** = neointermedeol, **46** = iso-β-bisabolol, **47** = α-bisabolol, **48** = tremeton, **49** = tetradecanoic acid (myristic acid), **50** = *n.i.* (fatty acid ester), **51** = *n.i.* (fatty acid ester), **52** = hexadecanoic acid (palmitic acid), **53** = *n.i.*, **54** = *n.i*, **55** = *n.i.*, **56** = *n.i.*, **57** = *n.i.*, **58** = campesterol, **59** = stigmasterol, and **60** = γ-sitosterol. *n.i.* = not identified.

The essential oil of *H. italicum* has an aromatic, mildly curry-like scent. Recent scientific studies have shown that it possesses numerous skin-related bioactivities that make it a desirable ingredient in natural cosmetics [53]. However, the components of essential oils are highly lipophilic substances. This limits their solubility in water and consequently their applicability in numerous formulations. While in the previous study dealing with extraction optimization [10] LC-MS analysis of phenolic components was performed (Table S1), in this study, the chemical composition of OPT-1 and OPT-2 extracts was further analyzed using GC-MS for the detection of the extracts’ volatile components. The results are provided in Figure 2 and Table S2. 

The chromatographs of OPT-1 and OPT-2 showed 60 peaks, out of which 52 have been tentatively identified using commercially available mass spectra libraries. Similar to the literature reports [5], the oil contained neryl acetate (**22**) in the largest relative abundance among the identified compounds, accounting for 2.75% and 2.18% in OPT-1 and OPT-2, respectively. Another compound presented in substantial amounts was *neo*-intermedeol (**45**) (1.86% in OPT-1 and 1.34%), a sesquiterpene, which has not been previously reported in *H. italicum*, but is quite abundant in an African species, *H. umbraculigerum.* Also relatively well represented in the extracts were β-selinene (**37**) (2.02 and 1.46 for OPT-1 and OPT-2, respectively), γ-curcumene (**35**) (1.17 and 1.22 for OPT-1 and OPT-2, respectively), 4,6,9-trimethyl-8-decene-3,5-dione, (italidione I) (**27**) (1.23 and 0.7 for OPT-1 and OPT-2, respectively), and nerol (**19**) (0.94 and 0.64 for OPT-1 and OPT-2, respectively) which have been reported before [5,15]. HP-β-CD was effective for the encapsulation of some non-polar molecules that may also be found in *H. italicum* essential oils such as, limonene, linalool, [24], and α-pinene [25]. However, to the best of our knowledge, this is the first attempt at the CD-assisted extraction of the bioactive volatiles of *H. italicum.* In addition, this is the first time that CD-assisted extraction has been found to be a successful approach for dissolving major compounds present in *H. italicum* essential oils such as neryl acetate, *neo*-intermedeol, β-selinene, γ-curcumene, and italidione I, rendering them soluble in water and thus suitable for use in water-based cosmetic products such as gels and lotions. 

In addition to volatile compounds, the extracts also contained substantial amounts of γ-sitosterol (1.16 and 2.03 for OPT-1 and OPT-2, respectively), as well as several other plant sterols, such as campesterol and stigmasterol, not usually reported as *H. italicum* essential oil constituents. The most probable cause of the observed quantitative discrepancies in this study is the method for the separation of lipophilic oil components. Namely, hydro-distillation is the most common technique for essential oil isolation, including *H. italicum* essential oils [15]. However, it is not suited for the separation of larger molecules from the plant matrix. In this work, on the other hand, the essential oil components were obtained by extraction. Thus, some of the observed differences in the composition are more likely to be connected to the isolation method than to the different plant chemistry. As the plant sterols may act as chemo-preventive, anti-inflammatory, antioxidant, antidiabetic, and anti-atherosclerotic agents [54], their presence in the plant extracts is likely to contribute to the beneficial cosmeceutical effects of the extracts. 

### 3.4. Antioxidant Activity of the H. italicum Extracts

Cosmetic products, such as creams and lotions, are rich in various substances sensitive to oxidative degradation, such as vitamins and polyunsaturated fatty acids. However, they are also intended to be stored for pronged periods of time both before and after their opening which makes them exposed to atmospheric oxygen. Thus, it is necessary for cosmetic products to contain antioxidants, free radical scavengers, and/or reducing agents that protect them against the oxidation that occurs during their storage and use [55]. Antioxidants in cosmeceutical formulations may also act as active ingredients by protecting dermal macromolecules against oxidative damage of the skin caused by environmental factors, such as UV radiation and free radicals [56,57]. The antioxidant activity of *H. italicum* extracts has been investigated using several methods. The influence of the prepared extracts on the free radicals (as modeled by DPPH free radicals), the reducing activity (as modeled by power in the reducing power assay), and the activity in heat-induced unsaturated fatty acid degradation in the β-carotene-linoleic acid system was investigated and compared with the activity of standard antioxidants, BHA and ascorbic acid. The essential oil and the extracts of *H. italicum* displayed notable antiradical activity in the DPPH assay [6,8,15]. However, the reducing power and the activity in the β-carotene-linoleic acid assay have not been described so far. It is important to note that, in the assays performed in this study, the activity of the extracts and the standards are expressed in different measurement units (μL extract/mL and μg/mL, respectively). Thus, it was not possible to compare them directly. However, it is possible to regard the activity of the standards as volume equivalents of 1 mg/mL solutions. Having this in mind, the activity of the standard antioxidants in the performed assays was measured and reported for general comparison purposes.

**Figure 3:**
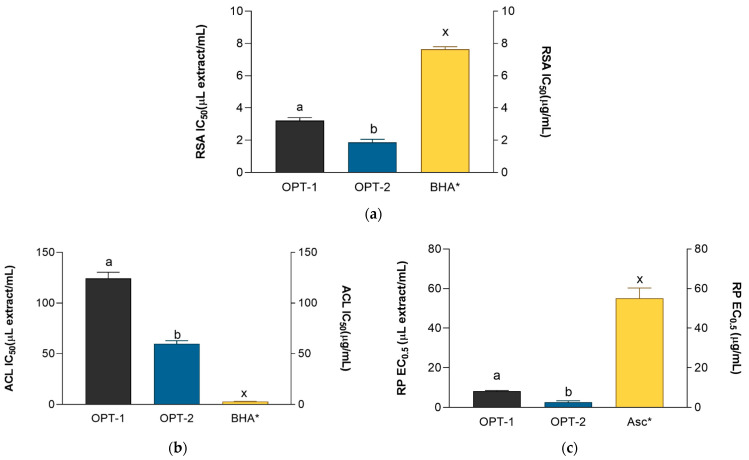
Antiradical activity (**a**), the activity in the β-carotene-linoleic acid assay (**b**), and the reducing power (**c**) of the OPT-1 and OPT-2 extracts (prepared as described in Section 2.6) and the positive controls, BHA (butylated hydroxyanisole) and Asc (ascorbic acid). ^a,b^ = differences between the extracts (Student’s *t*-test, *p* < 0.05). ^x^ = differences with the positive control (ANOVA followed by Dunnett’s post-test, *p* < 0.05). Columns not connected with the same letter are statistically different. The asterisk indicates that the unit is placed at the right ordinate.

Figure 3a–c depicts the results of the antioxidant assays performed in this work. Both extracts showed considerable activity in all the performed assays, but their activity depended on the assay. For example, both extracts inhibited the thermally induced degradation of the β-carotene-linoleic acid system. However, the activity of the extracts was notably weaker than the activity of the standard antioxidant, BHA. The antiradical activity and reducing power of the extracts, on the other hand, were outstanding. Both extracts were statistically stronger radical scavenger and reducing agents than the applied standard antioxidants, BHA and ascorbic acid (Dunnett’s test, *p* < 0.05). In all three performed assays, OPT-2, the extract containing a higher TP concentration, was a significantly stronger antioxidant than OPT-1 (Student’s *t*-test, *p* < 0.05). Thus, the large part of the observed activity is probably due to phenolic compounds, especially caffeic acid derivatives that were shown to be the main group of metabolites in OPT-1 and OPT-2 [10]. The antioxidant activity of caffeic acid is often stronger than the activity of well-established antioxidants, such as Trolox or ascorbic acid [8]. Additionally, caffeic acid is more stable than ascorbic acid and, unlike Trolox, is obtained from natural sources [58]. In addition, arzanol, a prenylated heterodimeric phloroglucinyl pyrone from *H. italicum* [8] that OPT-1 and OPT-2 extracts are rich in [10], shows antioxidant activity and is able to protect linoleic acid against free radical attack in assays of autoxidation and EDTA-mediated oxidation [14]. The *H. italicum* essential oil also displayed notable antioxidant properties in several studies [6,15,16], thus contributing to the observed activities of the extracts. Interestingly, the solvents also displayed a degree of antioxidant activity. The RSA IC_50_ values of the solvents used for the preparation of OPT-1 and OPT-2 were 56.07 ± 5.12 µL/mL and 65.72 ± 3.73 µL/mL, respectively. Similarly, the activities of the two solvents in the β-carotene-linoleic acid assay were 325.40 ± 62.25 µL/mL (solvent 1) and 347.69 ± 19.02 µL/mL (solvent 2). Finally, the RP EC_50_ values were 84.69 ± 4.16 µL/mL and 171.21 ± 10.31 µL/mL for solvent 1 and solvent 2, respectively. However, the activity of the solvents in the performed assays was insignificant in comparison with the activity of the corresponding extracts (Student’s *t*-test, *p* < 0.05).

### 3.5. Cosmeceutical Activity of the H. italicum Extracts

Contemporary cosmetic products and their ingredients are expected to act as functional ingredients and postpone or prevent processes that adversely influence skin health and appearance. As such, plant extracts and plant metabolites have a promising role in the development of new cosmeceutical products because they can inhibit the enzymes involved in the cellular aging process and the degradation of skin macromolecules. By doing so, they can delay the skin aging process and reduce its visible effects, such as skin dehydration, decreased elasticity, dark spots, and the formation of wrinkles [3]. In addition, plant metabolites may display their anti-ageing activity by hindering the development of skin changes caused by inflammation [37]. Previous research by this group demonstrated the excellent anti-collagenase and anti-ellastase properties of OPT-1 and OPT-2 extracts [10] (Table S1). In this study, hyaluronidase-, tyrosinase-, and LOX-inhibiting properties, as well as their activity in the ovalbumin coagulation-inhibiting assay, were investigated. Additionally, the ability of the extracts to absorb ultraviolet (UV) radiation was also assessed. As explained in the previous subsection, positive controls were tested for general comparison purposes, as it is possible to regard their activity as volume equivalents of 1 mg/mL solutions.

**Figure 4:**
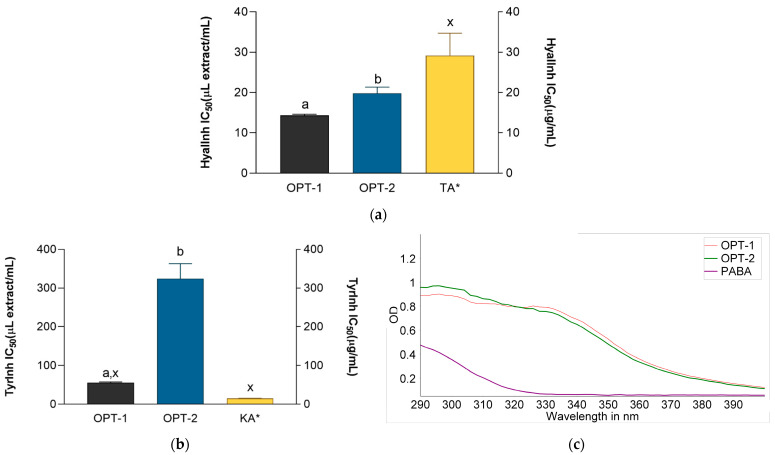
Hyaluronidase (**a**) and tyrosinase (**b**) inhibitory activity of the OPT-1 and OPT-2 extracts (prepared as described in Section 2.6) and positive controls KA (kojic acid) and TA (tannic acid) and UV-spectra of OPT-1 (red line), OPT-2 (blue line), and 1 mg/mL *p*-aminobenzoic acid (PABA) solution (**c**) in 1:64 dilution. ^a,b^ = differences between the extracts within a column (Student’s *t*-test, *p* < 0.05). ^x^ = differences with the positive control (ANOVA followed by Dunnett’s post-test, *p* < 0.05). Columns not connected with the same letter are statistically different. The asterisk indicates that the unit is placed at the right ordinate.

Reduced hydration of the skin leads to reduced turgor, resilience, and elasticity and the loss of youthful skin appearance. One of the most important macromolecules involved in skin hydration is hyaluronic acid, a polysaccharide that possesses an extreme water retaining capacity [59]. Hyaluronidase controls the turnover of hyaluronic acid in human skin. Its activity is increased in aging skin and skin affected by pathological processes, leading to the gradual loss of this polysaccharide in the skin and, consequentially, causing reduced skin tone and promoting the appearance of wrinkles [60]. Thus, inhibition of hyaluronidase leads to increased skin hydration and enhancement of skin appearance. As may be observed in Figure 4a, OPT-1 and OPT-2 were excellent hyaluronidase inhibitors, with their activity better than the activity of the tannic acid that was used as the positive control (ANOVA followed by Dunnett’s post-test, *p* < 0.05). In this assay, OPT-1 was the more active extract. The anti-hyaluronidase activity of *H. italicum* has not been reported before. However, it may be assumed that the activity observed in this study, at least in part, was caused by caffeic acid derivatives that OPT-1 and OPT-2 contain [10]. Namely, caffeic acid oligomers from *Clinopodium gracile* display hyaluronidase inhibitory activity. Such behavior is also characteristic of other caffeic acid derivatives, such as rosmarinic acid [61], as well as chicoric and caftaric acid [62]. Besides phenolic compounds, some essential oil components, such as geraniol [63] or linalool [64], may also display anti-hyaluronidase properties. Interestingly, the two solvents also displayed anti-hyaluronidase activity. While the activity of solvent 2 was quite low (268.48 ± 7.57 µL/mL), the activity of solvent 1 was relatively high (22.7 ± 1.41 µL/mL). Even then, the activity of the solvents was in both cases statistically lower than the activity of the corresponding extracts (Student’s *t*-test, *p* < 0.05); the relatively high activity of solvent 1 leads us to the conclusion that the extraction solvent also contributed to the observed activity of OPT-1. As the presence of lactic acid was the only difference between the two solvents, it may be postulated that the lactic acid inhibits the activity of hyaluronidase either through direct inhibition of the enzyme or through pH change. 

The anti-melanogenic potential of the prepared extracts was investigated through their anti-tyrosinase and UV-absorbing properties. Tyrosinase is the key enzyme in the synthesis of melanin, a pigment that determines skin color. It is a copper-containing polyphenol oxidase that converts L-tyrosine to L-DOPA and oxidizes L-DOPA to form dopachrome, which induces the production of melanin pigments. Melanin is responsible for the protection of skin from exposure to sunlight by absorbing harmful UV radiation. Despite the beneficial effects of melanin, its excessive production and accumulation leads to various skin disorders such as solar lentigo, melasma, and progressive hyperpigmentation. Tyrosinase inhibitors impede melanin synthesis and act as depigmenting agents in dermatological products [65,66]. The activity of the extracts, presented in Figure 4b, shows that, while both extracts exhibited tyrosinase inhibiting activity, the activity of the OPT-1 extract was substantially better and statistically equal to the activity of the standard tyrosinase inhibitor kojic acid (ANOVA followed by Dunnett’s post-test, *p* < 0.05). The higher activity of OPT-1 in this assay is mostly related to the presence of lactic acid, a compound known to suppress melanin formation by directly inhibiting tyrosinase activity, an effect independent of its acidic nature [67]. Namely, the activity of solvent 1 in this assay was 52.6 ± 2.43 µL/mL and did not statistically differ from the activity of OPT-1 (Student’s *t*-test, *p* < 0.05). On the other hand, the activity of solvent 2 was negligible (931.28 ± 136.1 µL/mL) indicating that the solvent did not participate in the activity of OPT-2. Thus, it seems that the use of HP-β-CD led to successful extraction of tyrosinase inhibitors from *H. italicum*. Methanolic extracts of *H. italicum* have previously been found to display notable anti-tyrosinase activity [68]. Numerous plant substances may display anti-tyrosinase activity. For example, *H. italicum* is rich in flavonoids [8], most notably quercetin derivatives, whose presence was observed in OPT-a and OPT-2 as well [10]. Quercetin derivatives display strong anti-tyrosinase properties [69]. In addition to quercetin, some essential oil components, such as linalool, have been demonstrated to display anti-tyrosinase activity in silico [70]. 

The risks associated with cumulative exposure or overexposure to solar UV radiation, including premature aging, skin damage, and even skin cancers, have been well documented. While UVB (290–320 nm) radiation is still considered to be the major factor responsible for most of the negative consequences of solar exposure, the harmful effects of UVA radiation (320–400 nm) have been increasingly documented [71,72]. Topical sunscreens contain UV filters that absorb UV radiation and protect the skin against the damage that it causes. Due to the perceived risks related to the use of synthetic sunscreens, plant-based ones are being increasingly developed [73]. The UV-absorbing properties of the investigated extracts were investigated spectrophotometrically [35] and compared to the absorbance properties of the topical sunscreen PABA. The absorbance spectra of the extracts (Figure 4c) revealed that both extracts absorb UV light with absorption maxima in the UVB and UVA ranges. The AUCs, expressed in arbitrary units (AU), for UVB (24.67 ± 4.20 AU and 27.06 ± 5.68 AU for OPT-1 and OPT-2, respectively) and UVA (37.74 ± 5.84 AU and 36.99 ± 7.33 AU for OPT-1 and OPT-2, respectively) did not statistically differ (Student’s *t*-test, *p* < 0.05) indicating that, when applied topically, both extracts may prevent photons from entering the skin. This infers not only additional anti-pigmentation properties but also the anti-carcinogenic potential [71] of the prepared *H. italicum* extracts. While the solvents used for extract preparation did not absorb UV light, the AUCs of PABA (7.61 ± 0.11 and 4.70 ± 0.11, for UVB and UVA region, respectively) were statistically lower than the AUCs of the extracts (ANOVA followed by Dunnett’s post-test, *p* < 0.05) indicating their excellent UV-absorbing properties. The observed effect is probably related to the presence of phenols in the prepared extracts. Namely, plant phenols may absorb the entire UVB spectrum of wavelengths, as well as a part of the UVA spectra, thus displaying sunscreen-like photoprotective properties [74].

**Figure 5:**
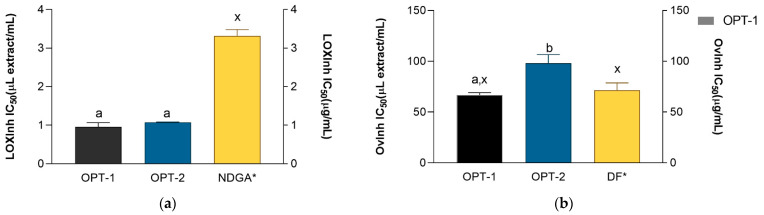
Lipoxygenase-(**a**) and ovalbumin coagulation inhibitory (**b**) activity of the OPT-1 and OPT-2 extracts (prepared as described in Section 2.6) and positive controls NDGA (nordihydroguaiaretic acid) and DF (diclofenac). ^a,b^ = differences between the extracts within a column (Student’s *t*-test, *p* < 0.05). ^x^ = differences with the positive control (ANOVA followed by Dunnett’s post-test, *p* < 0.05). Columns not connected with the same letter are statistically different. The asterisk indicates that the unit is placed at the right ordinate.

Inflammatory skin reactions include redness, rashes, edema, or defective physiological functioning of the skin [75]. Such skin appearance is characteristic of many skin diseases, such as atopic dermatitis or acne vulgaris [76]. Many of those effects are mediated by the LOX isoenzyme, which is responsible for inflammatory skin processes [77]. In addition, denaturation of tissue proteins is not only another cause but also characteristic of inflammatory processes [76]. For example, ultraviolet radiation produces protein denaturation, which in turn causes photoaging of the skin [78]. The plant extracts that suppress LOX activity and protein denaturation may be considered to have cosmeceutical and anti-aging activity because they hinder the development of inflammatory skin changes and related changes in skin appearance [37]. The investigated *H. italicum* extracts demonstrated excellent anti-inflammatory properties. Both extracts were effective LOX inhibitors (Figure 5a). While their activities were statistically equal (Student’s *t*-test, *p* < 0.05), both were also more active LOX inhibitors than NDGA (ANOVA followed by Dunnett’s test, *p* < 0.05). Both extracts were able to impede heat-induced ovalbumin coagulation (Figure 5b). However, better activity, statistically equal to the activity of the standard (Student’s *t*-test, *p* < 0.05), was displayed by OPT-2. It seems that the excellent anti-LOX activity of OPT-1 was mostly related to the presence of lactic acid. Namely, the activity of solvent 1 in this assay (0.81 ± 0.04 µL/mL) did not statistically differ from the activity of OPT-1. On the other hand, the activity of solvent 2 (1.41 ± 0.01 µL/mL) was still statistically lower than the activity of OPT-2 (Student’s *t*-test, *p* < 0.05) indicating significant activity by the extracted compounds in OPT-2. On the other hand, the two solvents display negligible activity in the ovalbumin assay. The anti-inflammatory properties of the investigated extracts are not surprising. *H. italicum* contains numerous caffeic acid derivatives [8], also present in OPT-1 and OPT-2, that demonstrated anti-protein coagulation activity in vitro [79]. In addition, arzanol, which OPT-1 and OPT 2 are rich in [10], inhibits eicosanoid biosynthesis by inhibiting 5-LOX [13]. Furthermore, 3,5-*O*-dicaffeoylquinic acid, another phenolic compound abundant in the prepared *H. italicum* extracts [10], is also a potent 5-LOX inhibitor [80]. Finally, according to previous studies, essential oil components, such as linalool, may also contribute to the anti-LOX activity [81].

### 3.6. Evaluation of the Extracts’ Influence on the Cell Viability

In order to determine the biocompatibility of the prepared extracts, the influence of the prepared *H. italicum* extracts on cell viability was tested on HaCaT cells, a long-lived, spontaneously immortalized human keratinocyte line able to differentiate in vitro [82]. Different concentrations (12.5–125 μL/mL) of the extracts, diluted in HBSS, were used to estimate the toxicity of the extract HaCaT cell cultures.

**Figure 6:**
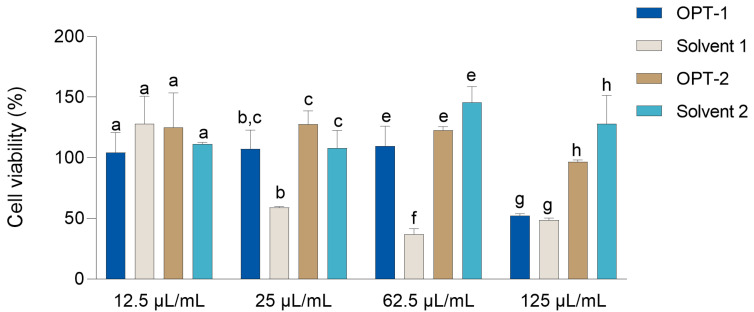
The influence of different dilutions of the OPT-1 and OPT-2 extracts (prepared as described in Section 2.6) on the survival of HaCaT cells. Cell survival is expressed as a percentage compared to cells treated with HBSS. The results are shown as the mean ± SD (*n* = 3). ^a^ = differences between the two extracts at a concentration of 12.5 μL/mL. ^b,c^ = differences between the two extracts at a concentration of 25 μL/mL. ^e,f^ = differences between the two extracts at a concentration of 62.5 μL/mL. ^g,h^ = differences between the extracts at a concentration of 125 μL/mL (series of Stutent’s *t*-tests, *p* < 0.05).

The results of the MTT test, presented in Figure 6, indicate that the extracts did not negatively affect viability HaCaT cells in concentrations up to 62.5 μL/mL (OPT-1) or even 250 μL/mL (OPT-2). Namely, the viability that the cells treated with 125 μL/mL OPT-2 displayed was 96.6 ± 1.6%. A previous study using a brine shrimp (*Artemia salina*) bioassay demonstrated that *H. italicum* essential oil is toxic in higher doses [15]. However, the high viability of the keratinocytes treated with the extracts recorded in this assay indicates their low toxicity and suitability for use in cosmetic formulations.

## 4. Conclusions

In continuation of previous efforts to prepare *H. italicum* extracts suitable for direct use in cosmetic products, further investigation of the chemical composition and cosmeceutical activity of OPT-1 (rich in phenolic acids) and OPT-2 (rich in total phenols and flavonoids) was performed. The prepared extracts were rich in phenolic compounds, including flavonoids and phenolic acids. In addition, HP-β-CD-assisted extraction was shown to be appropriate for the extraction and solubilization of the essential oil components of *H. italicum*. Both extracts displayed excellent antioxidant and cosmeceutical activity, which was, in the majority of the assays, better than the activity of the applied positive controls. OPT-2 displayed better antioxidant activity in the DPPH-radical scavenging assay, the β-carotene linoleic acid assay, and the reducing power assay. On the other hand, OPT-1 displayed better cosmeceutical properties as demonstrated by its hyaluronidase and tyrosinase inhibiting activity, as well as the ability to impede ovalbumin coagulation. In addition, the solvent used for the preparation of OPT-1 (containing lactic acid) contributed to the overall enzyme inhibitory activity of the extract. The extracts showed excellent biocompatibility with the human keratinocyte (HaCaT) cell line, assuring the status of excellent candidates for cosmeceutical product development.

## Figures and Tables

**Table 1:** Contents of selected metals in *H. italicum* aerial parts.

Metal	Concentration (Stem) (μg/g DW)	Concentration (Flower) (μg/g DW)
Cr	1.3 ± 0.2	1.6 ± 0.6
Cu	8.6 ± 0.2	13 ± 2.0
Fe	57 ± 5.0	82 ± 3.0
Ni	1.1 ± 0.1	1.9 ± 0.3
Pb	0.13 ± 0.02	0.07 ± 0.01
Se	0.08 ± 0.01	0.10 ± 0.01
Sr	4.3 ± 0.1	2.10 ± 0.1
Zn	19 ± 1.0	33 ± 1.0
As	<LOD	<LOD

DW = dry weight. <LOD = below the level of detection. The results are presented as the mean of three determinations ± SD.

## Data Availability

The data are contained within the article and Supplementary Materials.

## Supplementary Materials

The following supporting information can be downloaded at: https://www.mdpi.com/article/10.3390/antiox12040855/s1, Table S1. Summary of the research conducted on hydroxypropyl-β-cyclodextrin-assisted extraction and the resulting optimized extracts of *Helichrysum italicum*; Table S2. Volatile compounds in the *Helichrysum italicum* extracts as assessed by GC-MS analysis.
