# Supplementary material for: Hydroxypropyl-β-Cyclodextrin-Based Helichrysum italicum Extracts: Antioxidant and Cosmeceutical Activity and Biocompatibility
Source: Antioxidants (Basel). 2023 Apr 1;12(4):855. doi: 10.3390/antiox12040855 (PMC10135191; doi:10.3390/antiox12040855)
Supplement: Supplementary file 1 [file antioxidants-12-00855-s001.zip › antioxidants-2258754-supplementary.pdf]

antioxidants

Supplementary Materials

Hydroxypropyl-β-Cyclodextrin-Based *Helichrysum italicum* Extracts: Antioxidant, Cosmeceutical Activity and Biocompatibility with on HaCaT cells

Lejsa Jakupović [1], Ivana Bačić[2], Jasna Jablan[3], Eva Marguí [4], Marijan Marijan [1], Suzana Inić [3], Laura Nižić Nodilo [5], Anita Hafner [5], Marijana Zovko Končić [1,*]

[1] Department of Pharmacognosy, University of Zagreb Faculty of Pharmacy and Biochemistry, A. Kovačića 1, 10000 Zagreb, Croatia
[2] Forensic Science Centre "Ivan Vučetić", Ilica 335, 10000 Zagreb, Croatia
[3] Department of Analytical Chemistry, University of Zagreb Faculty of Pharmacy and Biochemistry, A. Kovačića 1, 10000 Zagreb, Croatia
[4] Department of Chemistry, Faculty of Sciences, University of Girona, C/ M. Aurèlia Capmany 69, 17003 Girona, Spain
[5] Department of Pharmaceutical Technology, University of Zagreb Faculty of Pharmacy and Biochemistry, A. Kovačića 1, 10000 Zagreb, Croatia
* Correspondence: mzovko@pharma.hr

Table S1. Summary of the research conducted on hydroxypropyl-β-cyclodextrin-assisted extraction and the resulting optimized extracts of *Helichrysum italicum*.

Previously published results [10]*	Results published in this work
**Extraction optimization**	
Preliminary solvent selection<br>Preliminary extraction kinetics<br>Extraction according to the 2-level factorial design<br>Extraction according to the Box-Behnken design	
**Chemical analysis**	
TP, TF, and TPA for optimization purposes<br>TP and TF in OPT 2, TPA in OPT 1<br>LC-MS	Determination of metal content in plant material<br>TP and TF in OPT 1, TPA in OPT 2<br>GC-MS
**Antioxidant activity**	
	Radical scavenging activity<br>Antioxidant activity in β-carotene-linoleic acid assay<br>reducing power
**Cosmeceutical activity**	
Elastase inhibitory activity<br>Collagenase inhibitory activity	Hyaluronidase inhibitory activity<br>Tyrosinase inhibitory activity<br>Measurement of UVA and UVB absorbing capabilities<br>Lipoxygenase inhibitory activity<br>Inhibition of heat-induced ovalbumin coagulation
**Biocompatibility**	
	Cell viability study on HaCaT cells

* = for reference list, see the main text; TP = Total phenol content, TPA= Total phenolic acid content, TF = Total flavonoid content. Optimized extracts: OPT-1 (rich in phenolic acids) and OPT-2 (rich in total phenols and flavonoids).

Table S2. Volatile compounds in the *Helichrysum italicum* extracts as assessed by GC-MS analysis.

No.	t_R	Compound	Content in OPT-1 (%)	Content in OPT-2 (%)
1	7.825	*α*-Pinene	0.35	0.23
2	9.433	*α*-Terpinene	0.03	0.03
3	9.484	*p*-Cymene	0.05	0.02
4	9.625	Eucalyptol	0.11	0.07
5	9.658	D-Limonene	0.10	0.06
6	10.170	*γ*-Terpinene	0.01	0.01
7	10.650	Cyclooctanone	-	0.05
8	10.708	*α*-Terpinolene	0.04	0.001
9	10.792	Linalool	0.78	0.47
10	11.033	Fenchol	0.07	0.03
11	11.392	Camphor (2-Bornanone)	0.07	0.02
12	11.442	L-Pinocarveol	0.07	0.03
13	11.675	2-methylbutyl angelate	0.08	0.04
14	11.700	Nerol oxyide	0.08	0.01
15	11.867	Endo-Borneol (Camphol)	0.19	0.07
16	12.033	4.6-dimethyloctane-3.5-dione	0.16	0.10
17	12.075	4-Terpineol	0.34	0.16
18	12.242	*α*-Terpineol	0.35	0.15
19	12.825	Nerol (geraniol)	0.94	0.64
20	13.267	Linalylacetate	0.08	0.07
21	13.767	4-Hydroxy-3-methylacetophenone	0.27	0.17
22	14.725	Neryl acetate	2.75	2.18
23	15.100	*α*-Muurolene	0.11	0.07
24	15.217	*α*-Copaene	0.12	0.10
25	15.592	*β*-Curcumene	0.11	0.08
26	15.675	trans-*α*-Bergamotene	0.06	0.04
27	15.717	4.6.9-Trimethyldec-8-en-3.5-dione (italidione I)	1.23	0.70
28	15.775	*β*-Caryophyllene	0.58	0.33
29	15.908	Neryl propionate	0.56	0.35
30	15.950	cis-*α*-Bergamotene	0.32	0.18
31	16.065	*n.i.*	1.14	1.36
32	16.208	Humulene	0.49	0.04
33	16.317	2.4.6.9-tetramethyldec-8-en-3.5-dione	0.89	0.57
34	16.425	*α*-Curcumene	0.96	0.56
35	16.450	*γ*-Curcumene	1.17	1.22
36	16.550	β-Sesquisabinene	0.07	0.10
37	16.600	β-Selinene	2.02	1.46
38	16.723	*γ*-Selinene	0.74	0.46
39	16.992	D-Cadinene	0.33	0.14
40	17.276	Dodecanoic acid (lauric acid)	0.17	0.26
41	17.692	Caryophyllene oxide	0.43	0.37

42	17.842	Guaiol	0.13	0.09
43	17.958	(1S.3αS.4S.5S.7αR.8R)-5-Isopropyl-1.7α-dimethyloctahydro-1H-1.4-methanoinden-8-ol	1.47	1.11
44	18.317	t-Cadinol	0.48	0.34
45	18.475	Neointermedeol	1.86	1.34
46	18.635	Iso-β-bisabolol	0.84	0.62
47	18.775	α-Bisabolol	0.47	0.47
48	19.042	Tremeton	0.88	0.33
49	19.542	Tetradecanoic acid (myristic acid)	0.44	0.66
50	19.792	*n.i.* (fatty acid ester)	-	0.87
51	19.867	*n.i.* (fatty acid ester)	0.14	0.57
52	21.608	Hexadecanoic acid (palmitic acid)	0.35	0.76
53	22.717	*n.i.*	7.95	4.78
54	23.600	*n.i.*	9.20	6.02
55	24.000	*n.i.*	3.12	2.2
56	24.800	*n.i.*	1.59	1.76
57	27.158	*n.i.*	2.64	6.45
58	32.583	Campesterol	0.54	0.53
59	32.975	Stigmasterol	0.57	0.73
60	33.758	γ-Sitosterol	1.61	2.03

n.i. = not identified.
